# Rationale and study design for empirical additional lesions for premature ventricular complex from the outflow tract: a multi-center, prospective randomized trial (EASE-PVC study)

**DOI:** 10.1007/s10840-022-01322-w

**Published:** 2022-08-06

**Authors:** Zhe Wang, Fangyi Xiao, Fu Yi, Chengzong Li, Long Chen, Cao Zou, Yuzhen Zhang, Yuegang Wang, Yuan Ji, Zhongbao Ruan, Wenzhi Shen, Linsheng Shi, Yumin Sun, Youquan Wei, Qiang Xu, Chen Wang, Weizhu Ju, Minglong Chen

**Affiliations:** 1 Department of Cardiology, The First Affiliated Hospital of Nanjing Medical University, Guangzhou Road 300, Nanjing, 210029 China; 2 Department of Cardiology, The First Affiliated Hospital of Wenzhou Medical University, Wenzhou, China; 3 Department of Cardiology, Xijing Hospital, Air Force Medical University, Xi’an, China; 4 Department of Cardiology, The Affiliated Hospital of Xuzhou Medical University, Xuzhou, China; 5 Department of Cardiology, School of Medicine, Zhongda Hospital, Southeast University, Nanjing, China; 6 Department of Cardiology, The First Affiliated Hospital of Soochow University, Soochow, China; 7 Department of Cardiology, Nanfang Hospital, Southern Medical University, Guangzhou, China; 8 Department of Cardiology, Changzhou No.2 People’s Hospital, The Affiliated Hospital of Nanjing Medical University, Changzhou, China; 9 Department of Cardiology, Taizhou People’s Hospital, Taizhou, China; 10 Department of Cardiology, Nanjing Drum Tower Hospital, Nanjing, China; 11 Department of Cardiology, The Second Affiliated Hospital of Nantong University, Nantong, China; 12 Department of Cardiology, Jing’an District Centre Hospital of Shanghai, Fudan University, Shanghai, China; 13 Department of Cardiology, The First Affiliated Yijishan Hospital of Wannan Medical College, Wuhu, China; 14 Department of Cardiology, Zhejiang Provincial People’s Hospital, Hangzhou Medical College, Hangzhou, China

**Keywords:** Premature ventricular complex, Catheter ablation, Outflow tract

## Abstract

**Background:**

Late recurrence after ablation remains a significant issue in patients with premature ventricular complexes (PVCs) who undergo catheter ablation. In this study, we aimed to test the hypothesis that empirical additional ablation (EAA) would improve the long-term control of PVCs from outflow tracts (OT-PVCs) compared with the approach of limited single point ablation at the assumptive location.

**Methods:**

EASE-PVC study (ChiCTR2200055340) is a prospective multi-center, randomized, and controlled trial designed to assess the effectiveness and safety of empirical additional ablation in patients with OT-PVCs. After successful elimination of OT-PVCs, the patients will be randomized into two groups. In patients randomized to the EAA group, additional lesion applications at sites surrounding the successful ablation site will be delivered empirically. For patients randomized to the control group, no additional empiric ablation will be performed around the successful ablation site. The primary endpoint will be freedom from PVC recurrence at 3 months following ablation, without antiarrhythmic drug therapy.

**Conclusions:**

The EASE-PVC study is designed to compare the effectiveness and safety of two different strategies for ablation in patients with OT-PVCs, namely empirical additional ablation strategy versus conventional single point ablation strategy. This prospective, multi-center, and randomized controlled trial, with comparative data evaluating procedural and long-term follow-up results, aims to elucidate the superiority of empirical additional ablation for the long-term control of OT-PVCs compared with the traditional single point ablation strategy.

**Clinical trial registration:**

Chinese Clinical Trials Registry Identifier: ChiCTR2200055340.

## Background

Catheter ablation has been established as an important treatment strategy for idiopathic premature ventricular complexes (PVCs). Success rates with PVC ablation can range from 50 to > 90%, and can vary based on PVC sites of origin, electrophysiological characteristics, ablation techniques, as well as operator experience [1]. Among the different reported locations, PVCs originating from the ventricular outflow tract (OT-PVCs) are the most commonly seen, and similarly have been the most studied [2–5].

The electrophysiological mechanism of OT-PVCs is thought to be firing from a focal origin resulting from the triggered activity or enhanced automaticity [6]. Theoretically, focal ablation with limited ablation at the assumptive origin, as determined by activation mapping or pace mapping, should destroy the tissue and eliminate the PVCs. However, in real practice, inaccuracies with mapping and catheter movement during ablation may result in inadequate lesions, leading to PVC recurrence. To compensate for these “imperfect” lesions, some operators empirically expand the lesion by burning at sites surrounding the “bull’s eye,” though, the value and safety of delivering empiric additional lesions (insurance burns) remain unproven.

In this study, we aim to test the hypothesis that empirical lesions would improve the long-term control of OT-PVCs compared with the strategy of single point burning at the assumptive location.

## Materials and methods

### Primary objective

This study is designed to compare the effectiveness and safety of empirical additional ablation strategy compared with the control group (strategy of single point burning) in patients with OT-PVCs undergoing first-time PVC ablation.

### Study design

EASE-PVC (ChiCTR2200055340) is a prospective multi-center, randomized, and controlled trial designed to assess the effectiveness and safety of empirical additional ablation in patients with OT-PVCs. Patients with structural heart disease (including ischemic heart disease, dilated cardiomyopathy, hypertrophic cardiomyopathy, left ventricular ejection fraction (LVEF) < 40%, NYHA III-IV status, history of cardiac surgery) and serious hepatic or renal dysfunction are excluded from enrollment. Additionally, individuals with a prior ablation for PVCs will also be excluded. The inclusion and exclusion criteria are listed in Table 1. Central randomization will be assigned in a 1:1 fashion between empirical additional ablation (EAA group) and “bull’s eye” ablation (control group).

**Table 1:** Inclusion criteria and exclusion criteria

Inclusion criteria
Age 18–75 years old
PVCs from outflow tract
Symptoms could not be controlled by drugs
Exclusion criteria
Previous heart surgery or any interventional therapy
Structural heart disease
Ischemic heart disease, Dilated cardiomyopathy, Hypertrophic cardiomyopathy, LVEF < 40%, NYHA III-IV status
Serious hepatica or renal dysfunction (AST/ALT > three time of upper limit; SCr > 3.5 mg/dl or Ccr < 30 ml/min)
Had accepted ablation procedure for PVCs
Expectation of life < 1 year
Female during pregnancy or at lactation period

***PVCs***, premature ventricular complexes; ***LVEF***, left ventricular ejection fraction; ***AST***, aspartate aminotransferase; ***ALT***, alanine aminotransferase; ***SCr***, serum creatinine; ***Ccr***, creatinine clearance

### Procedural approach

All antiarrhythmic drugs except amiodarone will be held for at least 5 half-lives before ablation. Amiodarone will be held for at least one half month. All procedures will be performed under local anesthesia.

A 6F quadripolar and a decapolar catheter will be advanced into the right ventricle and coronary sinus, respectively. Intravenous isoproterenol challenge and programmed right ventricular stimulation will be used to provoke the PVCs in the absence of PVCs at baseline.

**Fig. 1:**
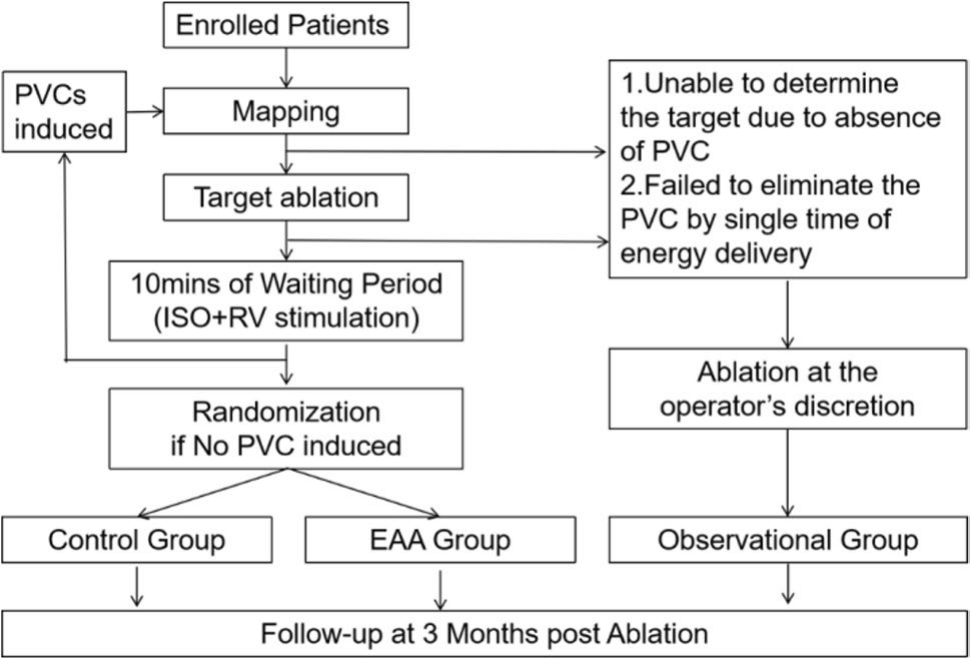
Study flow diagrams. PVCs: premature ventricular complexes; ISO: isoproterenol; RV: Right ventricular; EAA group: empirical additional ablation; Control group: “bull’s eye” ablation

Three-dimensional mapping will be performed to identify the earliest activation site of PVCs using a 3-dimensional mapping system (CARTO 3, Biosense-Webster Inc., Diamond Bar, CA, USA) system. Pace mapping using CARTO’s PASO module will be performed to identify the target only when PVCs are too infrequent to permit adequate activation mapping. After identifying the target, ablation will be attempted. Ablation will be performed with an open-irrigated tip catheter (Thermocool SmartTouch SF, Biosense Webster Inc. or Thermocool Smart Touch, Biosense Webster, Inc.). If the ablation effectively eliminates the PVCs, a single ablation lesion will be delivered for 60–90 s. Following elimination of the PVC, the surface electrocardiogram will be monitored for at least 10 min to assess for PVC recurrence, followed by a challenge of isoproterenol infusion (2–4 µg/kg·min) and standard programmed ventricular stimulation. If no PVCs are seen at this point, the patient will undergo randomization in a 1:1 manner into either the EASE group or control group. Randomization will be performed by an independent party not involved in the study who will open a sealed envelope. For patients randomized to the EASE group, additional lesion applications will be delivered at sites surrounding the “bull’s eye,” namely the previously effectively ablated point. The number and location of additional lesions will be left to the operator’s discretion based on actual technical and procedural considerations. Typically, 6 lesions will be delivered for consolidation adjacent to the successful ablation site (typically to the outflow tracts and above and below the adjacent pulmonic or aortic valve). Additional empiric ablation lesions will only be permitted in the same chamber. For example, if a PVC is eliminated with ablation from the septal RVOT, additional empiric ablation lesions will only be allowable from the RV or above the pulmonic valve, and left-sided lesions from the aortic cusps will not be permitted. In patients randomized to the control group, no additional empiric ablation will be delivered after the waiting period, and the procedure will be ended. If ablation fails to eliminate the PVCs, or if PVCs recur during the 10 min washout period, repeated mapping and ablation will be performed. In the following two situations, patients will not be randomized and will be categorized into the observational group: (1) The PVCs disappear spontaneously during the procedure and the site of origin or ablation effect are unclear; (2) The PVCs cannot be eliminated by repeated ablation (based on operator’s judgement). A flow-chart summarizing the study protocol has been provided in Fig. 1. In both groups, detailed ablation parameters and results will be recorded.

Generally, the maximum ablation power will be adjusted from 30 to 40 w at the discretion of the ablator, with temperature limit of 43 °C and irrigation rate of 17–30 ml/min. In the EASE group, additional ablations will be performed targeting the surrounding area close to the “bull’s eye.” If the successful ablation point was below the pulmonary or aortic valve, the corresponding area above the valve will be allowed to be targeted with empiric lesions, and vice versa.

### Follow-up

After ablation, anti-arrhythmic drugs will be discontinued, and no antiarrhythmic drugs will be allowed during follow-up. All patients will undergo 24-h Holter monitoring at 3 months after the procedure to assess PVC burden.

### Study endpoints

The primary endpoint will be freedom from PVC recurrence (defined as a ≥ 80% reduction of PVC burden) at 3 months following ablation, without antiarrhythmic drug therapy.

Secondary endpoints will include the following: (1) PVC burden at 3 months after ablation; (2) any peri-procedural complications.

### Statistical analysis

Based on the previous studies and our experience [7–9], the expected success rate will be 85% in the control group and 95% in empirical additional ablation. A sample size of 282 subjects (141 in each arm) is likely to be sufficient to detect a clinically important difference between groups using Pearson Chi-square test of proportions with 80% power and alpha of 0.025. Considering that approximately 10% of patients are expected to enter into the observational group based on experience, and accounting for a dropout rate of 1%, we calculate that 315 patients will be needed in this study. Accordingly, competitive enrollment will be initiated at each center. The patients will be randomly assigned to two groups by using permuted blocks with randomized block sizes 2 or 4 to ensure equal numbers in both groups. Randomized assignments will be placed in sealed envelopes and numbered consecutively. Random assignment codes will be generated by statistics professionals using the R version 4.1.1. Continuous variables will be expressed as the means ± standard deviations (normally distributed variables) or medians [25th and 75th percentiles]. Normal distribution will be assessed with the Shapiro–Wilk test. Categorical variables will be expressed as numbers and percentages. Chi-square testing will be used for inter-group comparison of success rate of surgery and a *P* value < 0.05 will be considered statistically significant. Statistical analyses will be performed using R version 4.1.1.

### Study organization and status

The study is a trial organized by the First Affiliated Hospital with Nanjing Medical University, Nanjing, China, and 18 centers in China will participate in this trial. The study was approved by the Ethics Committee of the First Affiliated Hospital of Nanjing Medical University (2021-SR-384.A1, Jiangsu Province Hospital) and institutional approval will be obtained from all centers before enrollment. This trial is sponsored by Beijing Xinlian Zhicheng Cardiovascular Health Public Welfare Foundation. Submission to the Chinese Clinical Trial Registry (http://www.chictr.org.cn) was posted on 01/07/2022, and the first patient was randomized on 10/28/2021. Completion of the study is expected by the end of 2022.

## Discussion

Catheter ablation was introduced into the clinical practice for the treatment of OT-PVCs over two decades ago [10]. There is a wide consensus that an extra heart beat is caused by one time of electrical impulse from a focus formed by a cluster of cells with abnormal automaticity [11]. Consequently, the ablation strategy for these idiopathic ventricular arrhythmias is to find the site exhibiting the earliest activation, or a site which demonstrates a perfect match compared with the clinical arrhythmia. These sites felt to represent surrogates of PVC origin. As so, factors influencing the process of mapping will impact the acute and long-term efficacy of PVC ablation. Occasionally, when there is a paucity of PVCs at the beginning of an ablation procedure, activation mapping might not be able to be performed, and in these situations ablation success rates may be lower — independent of the site of origin [12]. Additionally, inaccuracies with mapping may lead to ablation targeting incorrect sites, which may result in remote PVC relapse. Furthermore, slight movement during radiofrequency energy delivery, which is inevitable to some extent, may result in inadequate lesions. Based on these speculations, we hypothesized that empirical additional lesion applications surrounding the index ablation site will compensate the limitations of inaccurate mapping and inadequate lesions, thus improving the long-term control of PVCs.

To elucidate the “net efficacy” of the additional ablation lesions, we set a “blanking period” in each case after a lesion successfully eliminates of the PVC. The “blanking period” is indeed a standard and regularly used protocol to ensure an adequate and effective lesion, and includes 10 min of waiting period and a standard provocation protocol of drug challenge and right ventricular stimulation. Only those patients who demonstrate no PVCs after the “blanking period” will be randomized for the study. However, some situations will be excluded from the randomization. The first is the paucity of PVCs during mapping process, as this will result in difficulty in determining the target. The second situation is in cases where PVCs are unable to be eliminated with ablation. These two situations will be based on operator’s discretion and these patients will be categorized as “observational group” accordingly and their procedural data will be recorded.

To explore the association between the intra-procedure electrophysiological features and long-term control of PVCs, a new electroanatomic model (unpublished data from the First Affiliated Hospital with Nanjing Medical University) for the target electrogram will also be employed into this study. In that model, the characteristics of the target potential, including the prematurity and the morphological features, will be analyzed and correlated to the acute and long-term successful rate of OT-PVCs.

The additional benefit of quantitative ablation in the catheter ablation of PVCs remains uncertain [13–15]. Several studies have shown there to be no additional benefit of contact force sensing technology with regards to acute and long-term success rate of PVCs ablation, while another retrospective study showed that ablation index may be a predictor of long-term efficacy of PVCs ablation [16]. To date, the experience of quantitative ablation for ventricular arrhythmia ablation remains insufficient. Considering the fact that there is no consistent AI recommendation for the treatment of PVC ablation, the study will not introduce AI into the lesion evaluation; however, a contact force range of 5–20 g is set to minimize the ablation variance.

The study focuses on OT-PVCs ablation, whether the result could be extrapolated to the PVCs from other sites needs further studies.

## Conclusions

The EASE-PVC study is designed to compare the effectiveness and safety of two different strategies for ablation in patients with RVOT-PVC, namely empirical additional ablation strategy versus conventional single point ablation strategy. This prospective, multi-center, and randomized controlled trial, with comparative data evaluating procedural and long-term follow-up results, aims to elucidate the superiority and safety of empirical additional ablation for the long-term control of OT-PVCs compared with the traditional single point ablation strategy.
